# Analysis of MicroRNAs Associated With Carotid Atherosclerotic Plaque Rupture With Thrombosis

**DOI:** 10.3389/fgene.2021.599350

**Published:** 2021-02-12

**Authors:** Peng Nie, Fan Yang, Fang Wan, Shuxuan Jin, Jun Pu

**Affiliations:** Division of Cardiology, Renji Hospital, Shanghai Jiao Tong University School of Medicine, Shanghai, China

**Keywords:** atherosclerosis, plaque rupture, apoe knockout mice, microRNA 3, thrombosis

## Abstract

Atherosclerosis is a progressive vascular wall inflammatory disease, and the rupture of atherosclerotic vulnerable plaques is the leading cause of morbidity and mortality worldwide. This study intended to explore the potential mechanisms behind plaque rupture and thrombosis in ApoE knockout mice. The spontaneous plaque rupture models were established, and left carotid artery tissues at different time points (1-, 2-, 4-, 6-, 8-, 12-, and 16-week post-surgery) were collected. By the extent of plaque rupture, plaque was defined as (1) control groups, (2) atherosclerotic plaque group, and (3) plaque rupture group. Macrophage (CD68), MMP-8, and MMP-13 activities were measured by immunofluorescence. Cytokines and inflammatory markers were measured by ELISA. The left carotid artery sample tissue was collected to evaluate the miRNAs expression level by miRNA-microarray. Bioinformatic analyses were conducted at three levels: (2) vs. (1), (3) vs. (2), and again in seven time series analysis. The plaque rupture with thrombus and intraplaque hemorrhage results peaked at 8 weeks and decreased thereafter. Similar trends were seen in the number of plaque macrophages and lipids, the expression of matrix metalloproteinase, and the atherosclerotic and plasma cytokine levels. MiRNA-microarray showed that miR-322-5p and miR-206-3p were specifically upregulated in the atherosclerotic plaque group compared with those in the control group. Meanwhile, miR-466h-5p was specifically upregulated in the plaque rupture group compared with the atherosclerotic plaque group. The highest incidence of plaque rupture and thrombosis occurred at 8 weeks post-surgery. miR-322-5p and miR-206-3p may be associated with the formation of atherosclerotic plaques. miR-466h-5p may promote atherosclerotic plaque rupture via apoptosis-related pathways.

## Introduction

Atherosclerosis is a chronically progressive vascular wall inflammatory disease (Taleb, 2016). Over time, multiple subclinical cellular events cause the development of vulnerable, unstable atherosclerotic lesions. This leads to the rupture of atherosclerotic plaques and, ultimately, to catastrophic clinical manifestation of ischemic stroke or myocardial infarction (Chen et al., 2016). It has been reported that acute cardiovascular and cerebrovascular events caused by rupture of atherosclerotic vulnerable plaques are the leading cause of morbidity and mortality worldwide (Barrett, 2020). Thus, it is important to study the underlying mechanisms of atherosclerotic plaque rupture.

The features of vulnerable plaques include thin fiber caps, large lipid cores, plaque inflammation, neovascularization within the plaques, intraplaque hemorrhage, and intraluminal thrombosis (Cheruvu et al., 2007; Matter et al., 2009; Hansson et al., 2015). Presently, a significant obstacle to understanding the pathogenesis of plaque rupture is the lack of appropriate animal models to properly mimic the features of human atherosclerosis, especially plaque ruptures (Finn et al., 2010). In our previous study, we partially ligated the left renal artery and left common carotid artery (LCCA) to induce local stress and a continuously activated renin–angiotensin system in apolipoprotein E (ApoE) knockout mice. As such, a high incidence of spontaneous plaque rupture related to lumen thrombosis was generated successfully (Jin et al., 2012). This simple murine model (abbreviated as “R + C model”) not only recapitulated the pathophysiological processes of human plaque rupture but also generated rapid plaque progression. A successful animal model of plaque instability/rupture should respond to pharmacological agents known to reduce the risk of plaque ruptures in humans (Arca and Gaspardone, 2007; Jackson et al., 2007). Studies have demonstrated that after atorvastatin treatment, the plaque stability in R + C mouse models was improved independently of plasma cholesterol levels by modulating chemokine and receptor levels (Nie et al., 2014). Thus, the R + C model not only manifests the pathological characteristics of human plaques but also demonstrates similar reactivity to existing atherosclerotic drugs. This model may be useful for analyzing the mechanisms of action involved in atherosclerotic plaque instability.

MicroRNAs (miRNAs) are small non-coding RNAs (19–25 nt) that modulate protein synthesis by regulating mRNA expression (Bhat et al., 2016; Egea et al., 2017). As important regulators of pathophysiological processes, miRNAs provide a novel molecular perspective for the discovery of atherosclerotic mechanisms and therapeutic targets. Studies have demonstrated that miRNAs play important roles in the progression of atherosclerosis by regulating the expression of atherosclerosis-induced genes, as well as the expression of post-transcriptional genes in endothelial cells, smooth muscle cells, and macrophages (Feinberg and Moore, 2016; Maitrias et al., 2017). Nevertheless, the role of miRNAs in atherosclerotic plaque stability remains uncertain.

In this study, the R + C models were first established. Relevant samples at different time points after modeling were collected to investigate plaque occurrence and development, as well as to determine the optimal time points for plaque rupture and thrombosis. Additionally, through the preparation and analysis of the miRNA microarray, the potential pathophysiological mechanisms behind plaque rupture and thrombosis were also investigated.

## Materials and Methods

### Animal Experiments

All animal experiments were approved by the Ethics Committee of Renji Hospital affiliated to Shanghai Jiaotong University School of Medicine. ApoE knockout mice were purchased from the Jackson Laboratory (Bar Harbor, Maine, USA) and bred in the animal room with a standard rodent diet and tap water *ad libitum*. Eight-week-old mice were subjected to partial ligation of the left renal artery and LCCA to construct the R + C model. For time series experiments, mice were randomly assigned to seven groups: 1, 2, 4, 6, 8, 12, and 16 weeks post-surgery. To ensure that the studies were adequately powered, group sizes of at least 10 animals were used per group. For each time point, animals were euthanized, and the left common carotid artery tissue and plasma were collected. All surgeries were performed under a dissecting microscope.

### Surgical Procedures

#### Partial Carotid Ligation

Mice were anesthetized with isoflurane and oxygen (2.5% and 1 L/min) and maintained at 37°C on a heating pad. Partial ligation of the LCCA was performed, as described previously (Jin et al., 2012). Briefly, after blunt dissection to expose the distal branches of LCCA, blood flow was reduced by ligation (6-0 silk) of all branches of LCCA except for the left thyroid artery. After validation that blood flow was present, the incision was closed with a suture.

#### Partial Ligation of Left Renal Artery

Briefly, a spacer instead of a clip was chosen to ligate the left renal artery to generate a precise steady stenosis. Mice were anesthetized, and the left kidney was exposed through a small flank incision. After isolation by blunt dissection, the left renal artery was tied off (6-0 silk) along with a spacer (pin gauge, outer diameter = 0.11 mm). The pin gauge was subsequently pulled out leaving a tight stenosis in the artery. The kidney was then gently pushed back into the retroperitoneal cavity. The muscle layer and the skin incision were closed with sutures.

### Tissue Collection and Processing

Anesthesia was induced by intraperitoneal injection of xylazine (10 mg/kg) and ketamine (80 mg/kg) mixture. All mice were euthanized by cardiac puncture and then perfused with cold normal saline under physiological pressure. The left carotid artery was isolated and harvested under an operating microscope. Samples were stored in liquid nitrogen until needed.

Carotid tissues were optimal cutting temperature (OCT) embedded, and frozen sections were prepared. Each section of the carotid artery from the distal bifurcation was sectioned every 200 mm (2 mm in length).

### Morphological Analysis

Frozen sections were stained using hematoxylin–eosin (HE). Stable and vulnerable plaque morphologies were determined according to Virmani's criteria: grades 1–2 indicate stable plaques, and grades 3–6 indicate vulnerable plaques. Thin fiber caps (<3 cell layers) with large lipid cores (>40%) were defined as thin-cap fibroatheroma (TCFA). Plaque rupture was defined by an area of fibrous cap disruption whereby the overlying thrombus was in continuity with the underlying necrotic core (Schwartz et al., 2007). Intraplaque hemorrhage was defined as the deposition of blood products inside the plaque and was not necessarily associated with atherosclerotic plaque rupture (Schwartz et al., 2007).

Macrophage (CD68), MMP-8, and MMP-13-positively stained areas were determined by computer-assisted color-gated measurements and normalized to the total intimal surface area.

### Histology and Immunohistochemistry

Sections with the largest plaque burden in each carotid artery sample were selected for histology and immunohistochemistry analyses. Oil red O staining was used to evaluate the cholesteryl ester content of the plaques. Frozen sections were treated with 4% PFA and isopropanol, stained with oil red O working solution for 20 min, and then rinsed with 60% isopropanol for 5 s. After washing thoroughly in phosphate-buffered saline (PBS), the slides were mounted with glycerin for image acquisition.

### Vascular Collagen Content

Intima was evaluated using Sirius Red staining. Briefly, sections were stained using 0.1% Sirius Red working solution for 20 min and then rinsed with 0.5% acetic acid solution until background staining was removed. The slices were then dehydrated with anhydrous alcohol and mounted with glycerin. The stained samples were image captured using a Leica DM2500 optical microscope based on the fixed exposure settings. IPP image analysis software was used to analyze the percentage of lipid and collagen content in the plaque. Positive areas were determined by computer-assisted color-gated measurement and normalized to the total intimal surface area.

#### Immunofluorescence

LCCA segments containing atherosclerotic plaques were fixed in cold paraformaldehyde for 10 min, and serial cross-sections (5 mm) were triple-labeled with antibodies. Briefly, sections were blocked with 5% fetal bovine serum for 30 min, and incubated with primary antibodies against MMP-8, MMP13, TNF-α, IL-6, P62 (rabbit anti-mouse, diluted 1:100, Abcam), CD68 (rat anti-mouse, diluted 1:100, Millipore), and a-actin antibody (donkey anti-mouse, diluted 1:100, Millipore) overnight at 4°C. Secondary antibodies labeled with magenta fluorescence (goat anti-rat, 647 nm, Invitrogen), red fluorescence (donkey anti-mouse, 555 nm, Invitrogen), or green fluorescence (donkey anti-rabbit, 488 nm, Invitrogen) diluted 1:300 in bovine serum albumin (Invitrogen) were used to visualize the proteins of interest. After 60 min of incubation, the nuclei were stained with DAPI (4′,6-diamino-2-phenylindole, blue fluorescence), followed by image acquisition using a confocal microscope (Zeiss LSM 710). The positively stained areas were determined by computer-assisted color-gated measurements and then normalized to the total intimal surface area.

#### Analysis of Cytokines and Inflammatory Markers

Sandwich enzyme-linked immunosorbent assays (ELISAs) were used to measure high-sensitivity c-reactive protein (hs-CRP, ALPCO, Salem, NH, USA) and TNF-a (Bioscience, San Diego, CA, USA) from the plasma of experimental mice based on the manufacturer's instructions.

#### miRNA Expression Profile Microarray Preparation

miRNA microarray analysis was performed based on previously published protocols (Chen et al., 2015). Total RNA was extracted from left carotid artery samples [2, 4, 8, 12 weeks, and control groups (0 week)] and purified using the miRNeasy Mini Kit (QIAGEN, Shanghai, China) and RNase-Free DNase. RNA quality and quantity were analyzed using the Agilent 2100. FlashTag^TM^ Biotin RNA Labeling kit for Affymetrix GeneChip miRNA arrays were used for Poly(A) tailing and biotin labeling. The labeled miRNAs were incubated in a hybridization oven at 48°C for 16 h at 60 rpm, and then stained with the GeneChip Hybridization Wash and Staining Kit. GeneChips were scanned using the Hewlett-Packard GeneArray Scanner G3000 7G. Expression profile data were generated using the Affymetrix Expression Console software and normalized using the RMA method.

### miRNA Microarray Analysis

#### Differential Expression Analysis

Based on the miRNA microarray data and annotation file (miRBase database 20), the miRNA expression profile data were identified for differentially expressed miRNA (DEmis) screening using the R 3.4.3 package DEGseq (version 1.32.0). The DEmis included two parts: 8/12 weeks vs. control; 12/4 weeks vs. 8 weeks. The threshold was *p* < 0.05 or |log FC| > 1.5.

#### Time-Series Analysis

Short Time-Series Expression Miner (STEM, version 1.3.11) (Ernst and Bar-Joseph, 2006) was used for time-series analysis of miRNA expression patterns over time (0, 2, 4, 8, 12 weeks). The correlation coefficient of gene expression in each cluster was set to be higher than 0.7, the significance *p-*value was < 0.05, and the gene annotation source was Mouse. The miRNAs, which demonstrated a consistent trend (i.e., consistent color) were collected as miRNA sets.

#### miRNA-Target Regulatory Relation Prediction

In the Comparative Toxicogenomics Database (Davis et al., 2018), the genes directly related to diseases were searched with “atherosclerosis” as the keyword, and the disease genes with inference score > 100 were selected as the background genes. In the microT-CDS database, the DEmis and time-series related miRNAs were input into the target gene prediction module to analyze the mRNA–miRNA regulatory pairs. The mRNA–miRNA pairs with regulatory relation >0.95 were screened, and the miRNA regulatory network was constructed by combining the background genes.

#### Function Enrichment Analysis of miRNAs

The target genes of DEmis and time-series-related miRNAs were analyzed using the KEGG (Kanehisa and Goto, 2000) pathway and Gene Ontology (GO) (Ashburner et al., 2000) biological process (BP) enrichment analyses, using the ClusterProfiler R package (version 3.2.11) (Yu et al., 2012). The parameters of count ≥ 2 and *p* < 0.05 were used as significant thresholds.

#### Protein–Protein Interaction (PPI) Network Analysis

The interaction relations between target genes (coding proteins) of miRNAs were predicted and analyzed using the STRING (version 11.0) (Szklarczyk et al., 2014) database. The input gene set was the target genes of DEmis and time-series-related miRNAs, and the species was mus. In order to obtain more interaction relations, the parameter of PPI score was set to 0.4 (medium confidence). The network was constructed using Cytoscape (version 3.2.0) (Shannon et al., 2003).

#### Competing Endogenous RNA (ceRNA) Network Construction

DIANA-LncBase version 2 (Jin et al., 2012) was used to predict the miRNA-related lncRNAs. The lncRNA–miRNA pairs with regulatory relation > 0.99 were screened. Then the miRNA–target regulatory relation pairs were integrated with lncRNA–miRNA pairs to construct the ceRNA.

#### Literature Retrieval of the Key Target Gene

Based on the human–mice homologous genes indicated in various databases, the target genes in the ceRNA network were aligned with the human homologous genes for homology analysis. The literatures associated with “Atherosclerosis” and “Plaque” were summarized using the GenCLiP 2.0 (Wang et al., 2014) database.

### Statistical Analysis

Data are expressed as mean ± standard error of the mean (SEM). The Kolmogorov–Smirnov test was used to determine whether the data were normally distributed. Differences between the groups were compared using two-tailed Student's t-tests or one-way analysis of variance. Differences in the classification and occurrence of adverse events were analyzed by a chi-squared test. *P* < 0.05 were considered statistically significant. Statistical analysis was performed using SPSS version 17.0 software (SPSS Inc., Chicago, IL, USA) and GraphPad Prism 6.0 software.

## Results

### The Highest Incidence of Plaque Rupture and Thrombosis Occurred at 8 Weeks Post-surgery

**Figure 1:**
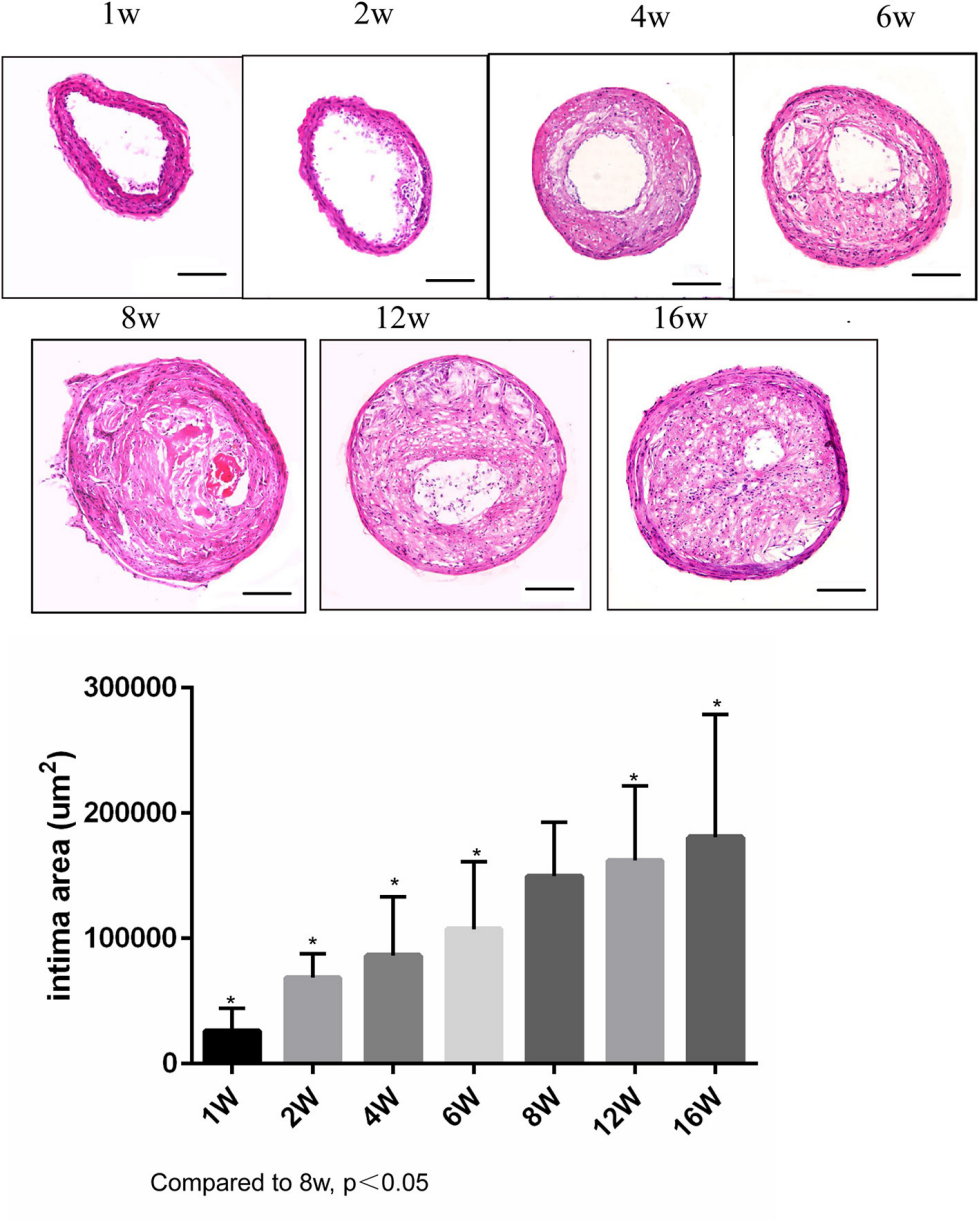
Lesion development in the carotid artery of apolipoprotein E (ApoE)^−/−^ mice undergoing R + C at 1, 2, 4, 6, 8, 12, and 16 weeks post-surgery. *P < 0.05 compared to that of 8 weeks. Images for magnifications are 200× (scale bar 100 μm).

ApoE knockout mice underwent R + C surgery and were then euthanized at 1, 2, 4, 6, 8, 12, and 16 weeks post-surgery. Plaque load and plaque morphology were then determined. As shown in Figure 1, plaque load increased with time. Compared with 8 weeks post-surgery, there was a significant increase in plaque load at 12 and 16 weeks (both *p* < 0.05). However, there was no statistically significant difference in plaque load between 12 and 16 weeks.

Plaque morphology was analyzed at 1–2 weeks post-surgery to observe the early lesion features for atherosclerosis. At 4–6 weeks, plaques gradually became vulnerable, with the highest incidence of vulnerable plaques occurring at 8 weeks. The incidence of plaque rupture with intraluminal thrombosis was 50%, of buried fibrous cap was 80%, and of plaque bleeding was 80%. At 12 and 16 weeks, although the plaque load increased, the detection rate for vulnerable plaques decreased (70 and 60%, respectively), and the incidence of plaque rupture was lower compared to the 8-week group. Therefore, the highest incidence rate of plaque rupture with thrombotic lesions in the R + C model was around 8 weeks post-surgery (Table 1).

**Table 1:** Lesion features at 1, 2, 4, 6, 8, 12, and 16 weeks post-surgery.

**Mice**	**Stable phenotype**	**Vulnerable phenotype**	**Intraplaque hemorrhage**	**Multilayer with discontinuity**	**Rupture with thrombus**
1w (*n =* 10)	100% (10)	0% (0)	0% (0)	0% (0)	0% (0)
2w (*n =* 10)	80% (8)	20% (0)	0% (0)	0% (0)	0% (0)
4w (*n =* 10)	40% (4)	60% (6)	20% (2)	20% (2)	10% (1)
6w (*n =* 10)	40% (4)	60% (6)	30% (3)	40% (4)	20% (2)
8w (*n =* 10)	10% (0)	90% (1)	80% (8)	80% (8)	50% (5)
12w (*n =* 10)	30% (0)	70% (7)	70% (7)	70% (7)	20% (2)
16w (*n =* 10)	40% (4)	60% (6)	60% (6)	60% (6)	0% (0)

### The Number of Plaque Macrophages and Lipids Were at Their Highest, While the Number of Smooth Muscle Cells, and Collagen Fibers Were at Their Lowest, at 8 Weeks Post-surgery

**Figure 2:**
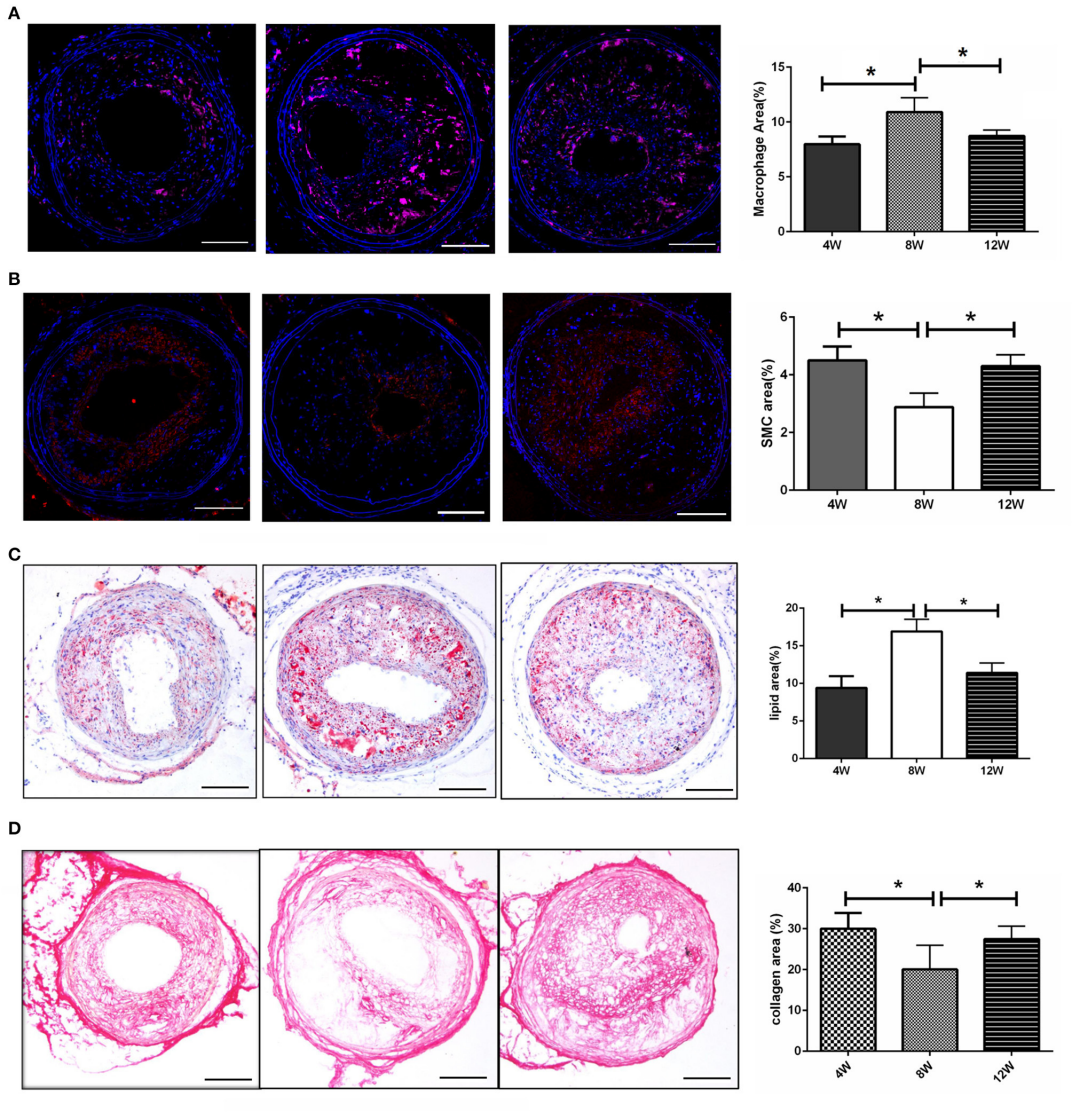
Morphology of atherosclerotic plaques. **(A)** The number of macrophages in the plaques. **(B)** The number of vascular smooth muscle cells in the plaques. **(C)** The lipid content in the plaques. **(D)** The collagen fiber content in the plaques. *P < 0.05 compared to that of 8 weeks. Images for magnifications are 200× (scale bar 100 μm).

To determine the mechanism of plaque rupture, the plaque components at 8-weeks post-surgery (when the incidence of plaque rupture was the highest) were analyzed. As shown in Figure 2A, the number of macrophages in the plaques of the 8-week group were significantly higher than that of the 4- and 12-week groups (both *p* < 0.05). In addition, the number of vascular smooth muscle cells in the 8-week group were significantly lower than those in the 4- and 12-week groups (both *p* < 0.05) (Figure 2B). Furthermore, the 8-week group had the highest plaque lipid content and the lowest collagen fiber content compared with the 4- and 12-week groups (all *p* < 0.05) (Figures 2C,D).

### The Expression of Matrix Metalloproteinase Was the Highest in the 8-Week Group

**Figure 3:**
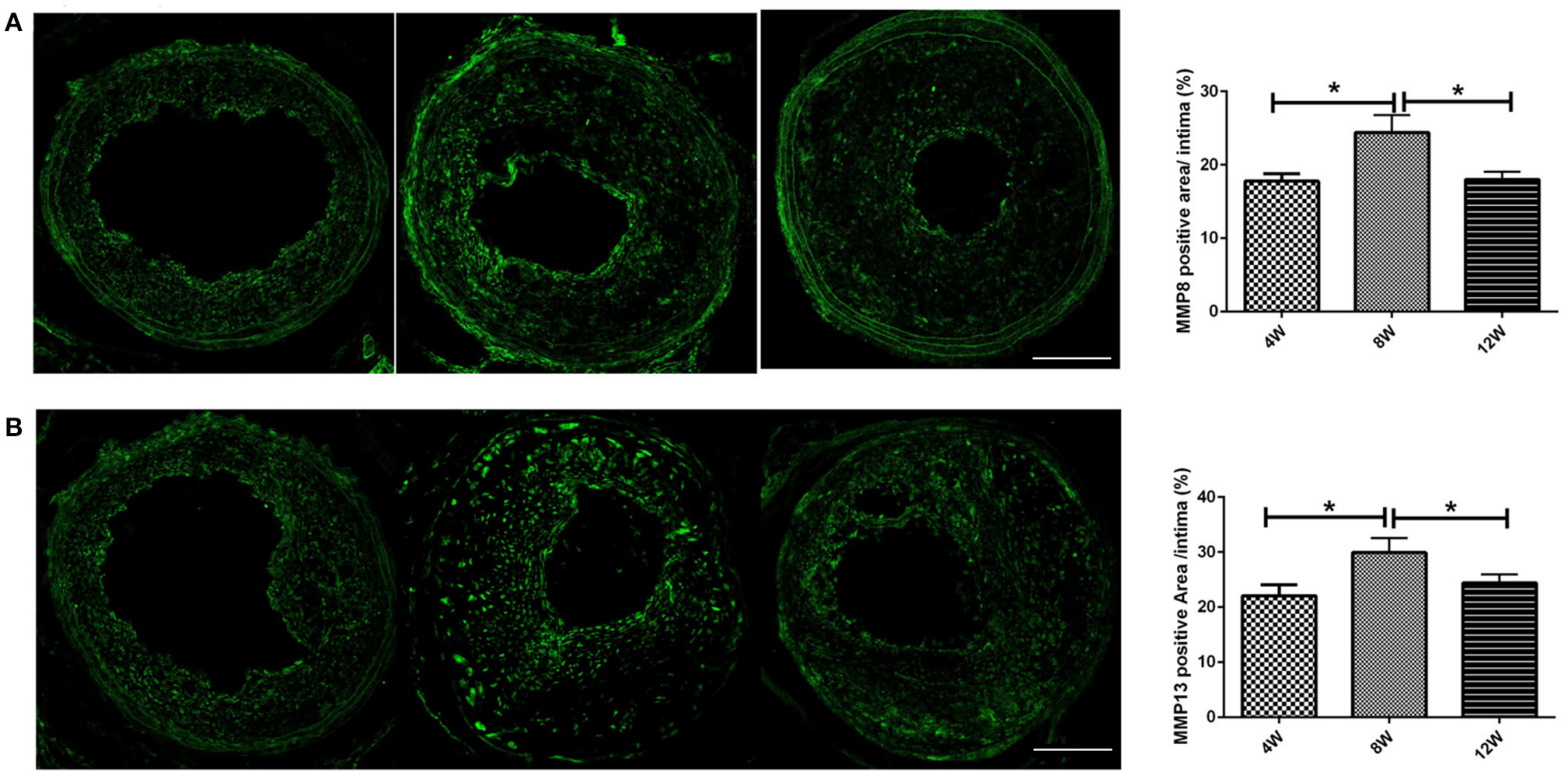
MMPs (**A**: MMP-8; **B**: MMP-13) expression of atherosclerotic plaques in ApoE^−/−^ mice. *P < 0.05 compared to that of 8 weeks. Images for magnifications are 200× (scale bar 100 μm).

MMP8 and MMP13 are important subtypes of matrix metalloproteinases, which play a key role in the formation of vulnerable plaques in ApoE knockout mice (Cheng et al., 2009; Levine et al., 2011). We analyzed the expression of MMP8 and MMP13 in the left common carotid plaque after R + C modeling. Both MMP8 and MMP13 had the highest expression levels in atherosclerotic plaques in the 8-week group compared with the 4- and 12-week groups (all *p* < 0.05) (Figures 3A,B).

### The Atherosclerotic and Plasma Cytokine Levels Were the Highest in the 8-Week Group

**Figure 4:**
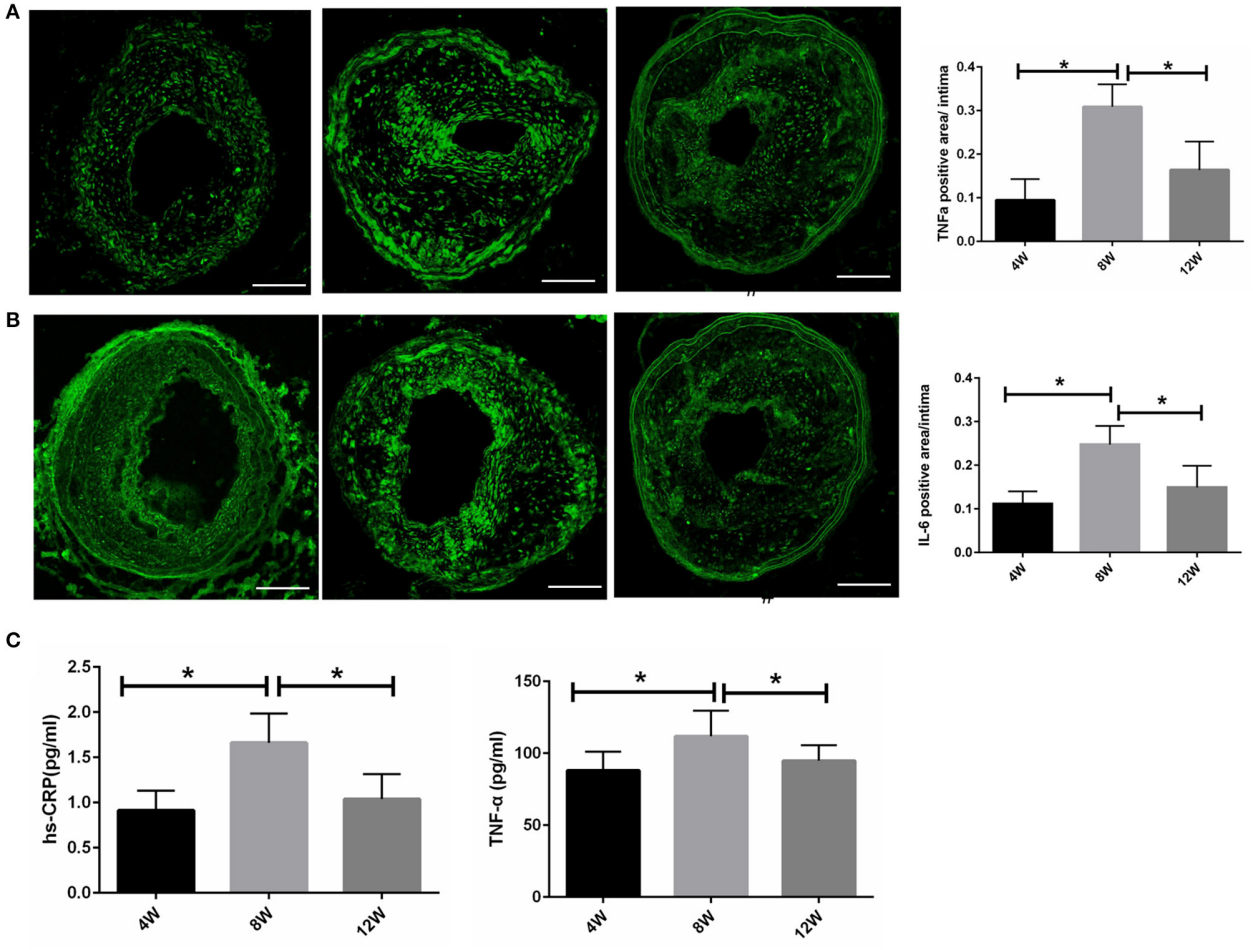
Atherosclerotic and plasma cytokine levels in ApoE^−/−^ mice. **(A,B)** Atherosclerotic TNF-α and IL-6 levels. **(C)** Plasma TNF-α and hs-CRP levels. *P < 0.05 compared to that of 8 weeks. Images for magnifications are 200× (scale bar 100 μm).

To analyze the differences in plaque and systemic inflammation in the 4-, 8-, and 12-week groups, atherosclerotic and plasma cytokine levels were detected. Immunohistochemical staining for TNF-α and IL-6 was performed on the LCCA segment with the largest plaque load for each mouse. Compared with the 4- and 12-week groups, TNF-α and IL-6 were at their highest levels in the 8-week group (all *p* < 0.05) (Figures 4A,B). Additionally, specific ELISA kits were used to evaluate plasma TNF-α and hs-CRP levels from each group. As shown in Figure 4C, TNF-α and hs-CRP were at their highest levels in the 8-week group compared with the 4- and 12-week groups (all *p* < 0.05).

### p62 Expression Levels in Plaque Macrophages Were the Lowest at 8 Weeks

**Figure 5:**
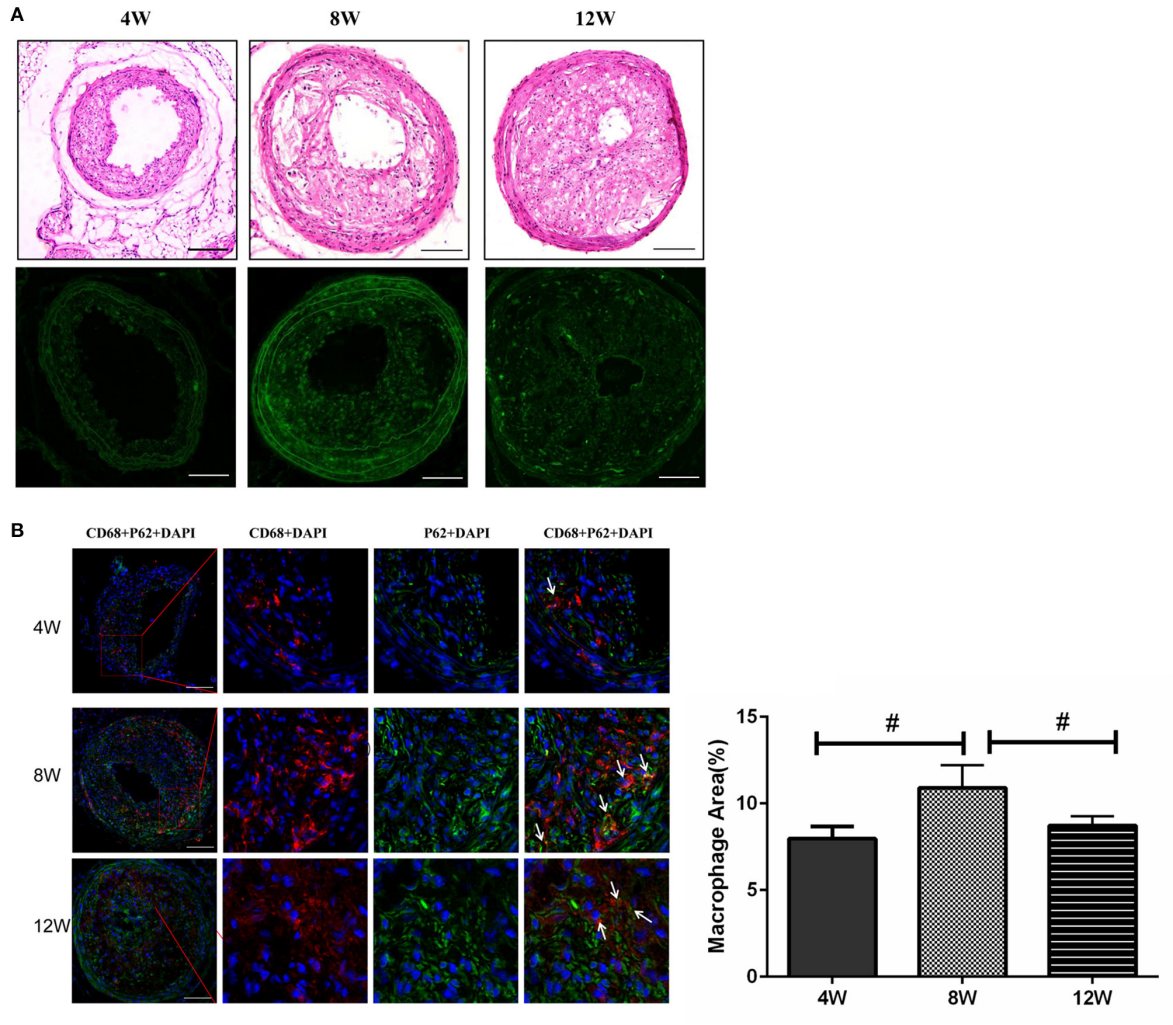
Macrophage autophagy in atherosclerotic plaques in ApoE^−/−^ mice. **(A)** The expression level of autophagy chaperone protein p62. **(B)** The number of p62 + CD68 cells. #*P* < 0.05 compared to that of 8 weeks. Images for magnifications are 200× (scale bar 100 μm).

It has been reported that plaque-stimulating factors can inhibit autophagy in macrophages, directly leading to an increase in vascular inflammatory response and disease progression (Cheng et al., 2009; Levine et al., 2011). Thus, we detected the expression level of autophagy chaperone protein p62 through immunofluorescence analysis. When lysosomal autophagy is blocked, it begins to aggregate in cells. Autophagy levels in macrophages for each group were evaluated by counting the number of p62 + CD68 + macrophages. The number of p62 + CD68 cells in the 8-week group was significantly higher than in the 4- and 12-week groups (both *p* < 0.05). This suggested that autophagy in macrophages was blocked in the 8-week group (Figure 5).

### miRNA Microarray Analysis

**Figure 6:**
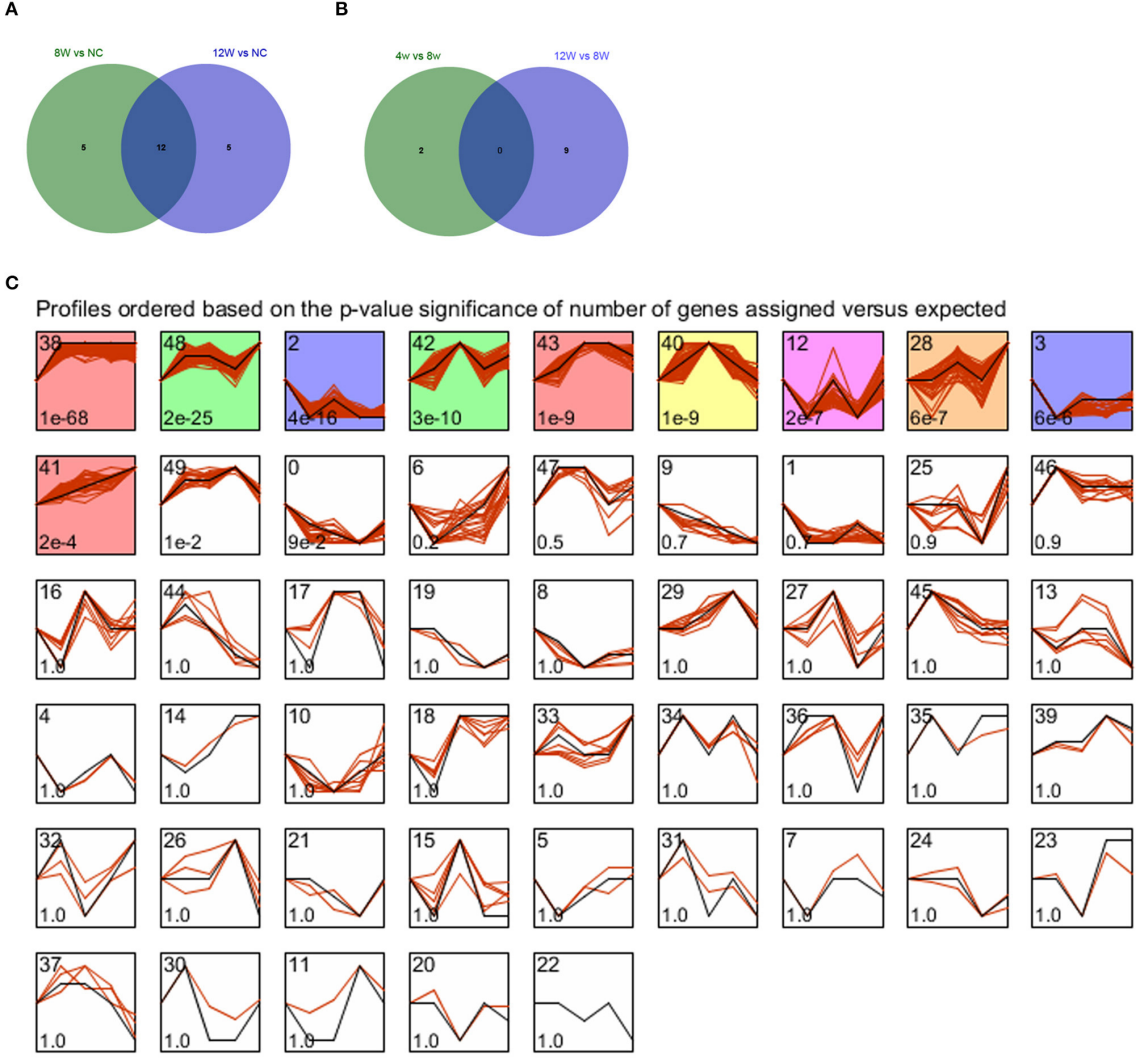
**(A,B)** VENN diagrams for atherosclerotic plaque (4- and 12-week) vs. control groups and plaque rupture group (8-weeks) vs. atherosclerotic plaque groups (4- and 12-week). **(C)** STEM clustering diagram. The black broken line in the square indicates the overall trend of the expression of all the genes in the gene set. The number in the lower left corner of the square represents the *p-*value of the significance of gene expression similarity in the clustering gene set. The values with color are significant, and the values with consistent color are consistent.

Compared with the control group, there were 17 DEmis in the 8- and 12-week groups, each. VENN analysis showed that there were 22 union set genes, such as miR-322-5p and miR-206-3p (Figure 6A). Compared with the 8-week group, there were, respectively, two and nine DEmis in the 4- and 12-week groups. VENN analysis showed that there were 11 union set genes, including miR-466h-5p, miR-144-5p, miR-742-5p, miR-184-5p, miR-703, miR-665-5p, miR-450a-5p, miR-186-5p, miR-450b-3p, miR-124-5p, and miR-743b-5p (Figure 6B).

All genes were subjected to STEM-based time series expression profiling analysis, and a total of 10 significantly enriched clusters were screened, as shown in Figure 6C. Consistent color indicates consistent trend, so we combined profile 38, 43, and 41 as a red-cluster. Profile 48 and 42 were combined as a green-cluster. Profiles 2 and 3 were combined as a purple cluster. The red cluster that demonstrated an uptrend in expression was selected for the following analysis.

**Figure 7:**
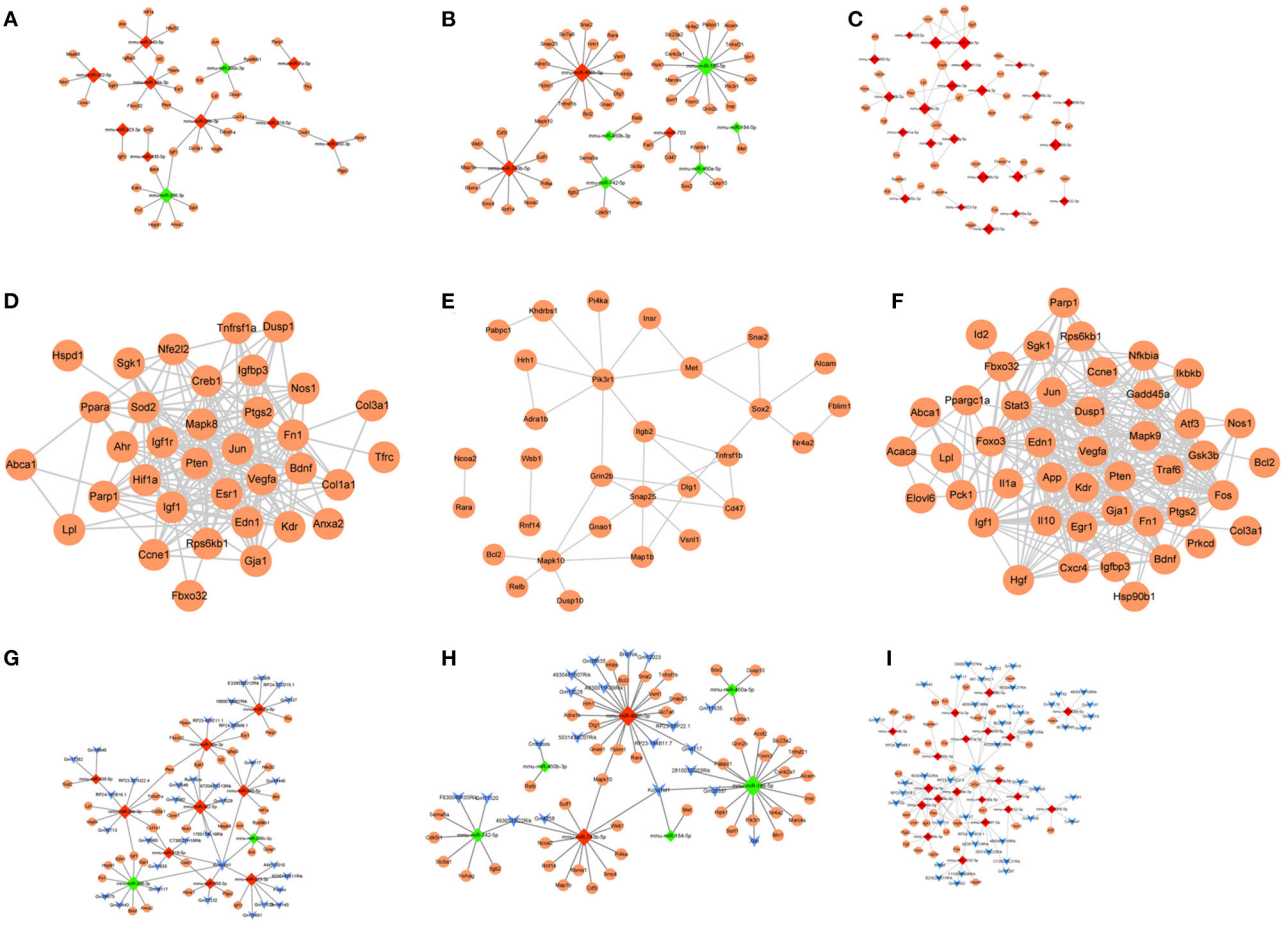
**(A–C)** The miRNA-target regulatory networks for atherosclerotic plaque (4- and 12-week) vs. control groups, plaque rupture group (8-weeks) vs. atherosclerotic plaque groups (4- and 12-week), and time series module. **(D–F)** The PPI networks for atherosclerotic plaque (4- and 12-week) vs. control groups, plaque rupture group (8-weeks) vs. atherosclerotic plaque groups (4- and 12-week), and time series module. **(G–I)** The ceRNA regulatory networks for atherosclerotic plaque (4- and 12-week) vs. control groups, plaque rupture group (8 weeks) vs. atherosclerotic plaque groups (4- and 12-week), and time series module. The circle is mRNA, the red rhombus is upregulated miRNA, the green rhombus is downregulated miRNA, and the blue inverted triangle is lncRNA.

miRNA–target prediction analysis showed that 43 miRNA–target regulatory pairs (11 miRNAs and 36 target genes) were predicted based on the union set miRNAs of atherosclerotic plaque (4- and 12-weeks) vs. control groups (Figure 7A). Additionally, 51 miRNA–target regulatory pairs (eight miRNAs and 50 target genes) were predicted based on the union set miRNAs of the plaque rupture group (8-weeks) vs. atherosclerotic plaque groups (4- and 12-weeks) (Figure 7B). For the time series-related miRNAs, 53 miRNA-target regulatory pairs were predicted, involving 23 miRNAs and 36 target genes (Figure 7C).

**Figure 8:**
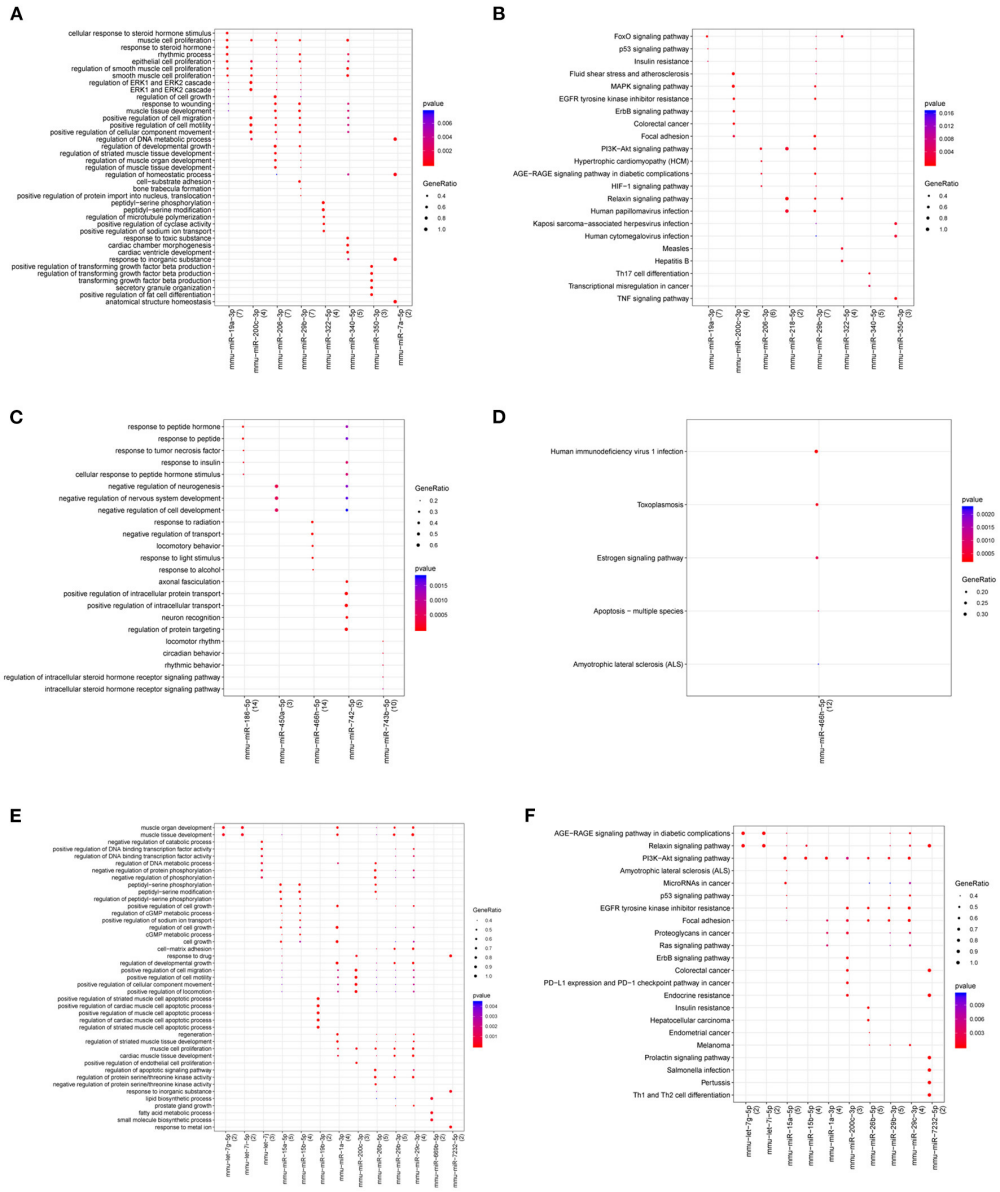
Gene Ontology (GO) and KEGG pathway for miRNAs in atherosclerotic plaque (4- and 12-week) vs. control groups **(A,B)**, plaque rupture group (8-week) vs. atherosclerotic plaque groups (4- and 12-week) **(C,D)**, and time series module **(E,F)**. The horizontal axis represents the miRNA, the vertical axis represents the significantly correlated GO and KEGG pathways, and the change of bubble color from blue to red represents the correlation significance from low to high. The dot size GeneRatio represents the proportion of genes. The larger the proportion of genes, the larger the proportion of this enrichment.

Functional prediction of miRNAs revealed that compared with the control group, 59 relevant pathways (such as PI3K-Akt signaling pathway, FoxO signaling pathway, fluid shear stress and atherosclerosis, and MAPK signaling pathway) and 1,050 BP functions (such as cellular response to steroid hormone stimulus, ERK1 and ERK2 cascade, anatomical structure homeostasis, and smooth muscle cell proliferation) were obtained in the atherosclerotic plaque group. Compared with the atherosclerotic plaque group, five pathways (human immunodeficiency virus 1 infection, estrogen signaling pathway, and apoptosis-multiple species) and 348 BP functions (response to peptide hormone and positive regulation of intracellular transport) were obtained in the plaque rupture group. Moreover, 124 relevant pathways (AGE-RAGE signaling pathway in diabetic complications, relaxin signaling pathway, and PI3K-Akt signaling pathway) and 1,077 BP functions (muscle organ development, and muscle tissue development) were obtained for the time series-related miRNAs (Figure 8).

For the constructed PPI networks, there were 35 nodes and 205 interaction pairs in atherosclerotic plaque (4- and 12-weeks) vs. control groups. In the plaque rupture group (8-weeks) vs. atherosclerotic plaque groups (4- and 12-week), the constructed network contained 30 nodes and 38 interaction pairs. Based on the time series-related target genes, the PPI network contained 36 nodes and 199 pairs of interaction relations (Figures 7D–F).

Based on the miRNA–target pairs, as well as the predicted 49 miRNA–lncRNA relation pairs, the ceRNA network (including 11 miRNAs, 36 mRNAs, 34 lncRNAs, and 85 ceRNA regulatory relation pairs) was constructed in atherosclerotic plaque vs. control groups. In plaque rupture group vs. atherosclerotic plaque groups, the ceRNA network consisted of seven miRNAs, 48 mRNAs, 20 lncRNAs, and 74 ceRNA relation pairs. In the time series module, the ceRNA network contained 20 miRNAs, 34 mRNAs, 45 lncRNA, and 196 ceRNA regulatory pairs (Figures 7G–I).

For 118 target genes in the ceRNA networks, literature retrieval was carried out according to the method described, and 24 genes, such as vascular endothelial growth factor A (*Vegfa*), insulin-like growth factor 1 (*Igf1*), and mitogen-activated protein kinase 8 (*Mapk8*), were found to be associated with “atherosclerosis” and “plaque” (Table 2).

**Table 2:** The literatures of target genes.

**Gene**	**Hit**	**Total**
PTGS2	9	27,618
IL10	8	43,716
VEGFA	7	50,679
IGF1	5	38,486
HSPD1	5	6,790
PARP1	3	9,025
ABCA1	3	2,755
PPARA	2	7,835
CXCR4	2	12,097
LPL	2	8,840
IL1A	1	9,772
FN1	1	761
IGFBP3	1	6,299
ESR1	1	17,047
GJA1	1	6,182
EGR1	1	4,021
SOD2	1	5,205
TNFRSF1A	1	3,829
SLC9A1	1	1,410
HRH1	1	1,417
APP	1	20,435
TRAF6	1	1,472
MAPK8	1	19,256
IGF1R	1	4,184

## Discussion

Atherosclerotic plaque rupture is the leading cause of atherosclerosis-related death. Understanding the pathophysiological mechanism of plaque rupture is of significance for the prevention and treatment of acute cardiovascular and cerebrovascular diseases caused by atherosclerotic plaque rupture (Bentzon et al., 2014). Presently, we constructed the R + C model and designed a time series study post R + C surgery, to examine the pathogenesis and optimal time point of plaque rupture and thrombosis.

The results showed that fatty streaks appeared at 1–2 weeks post R + C surgery. Unstable plaque morphology and rupture occurred after 4 weeks, and plaque rupture with thrombus and intraplaque hemorrhage peaked at 8 weeks. At 12–16 weeks, plaque burden increased with the increased incidence of buried fibrous caps, while the incidence of plaque rupture and thrombosis decreased. Our previous study demonstrated that activation of the renin–angiotensin–aldosterone system, local shear stress on the plaque, and high blood pressure, can promote plaque rupture (Jin et al., 2012). It has been reported that overexpression of collagenases and reduced collagen synthesis signaled by inflammatory mediators can impair the biomechanical integrity of the plaque's fibrous cap. Pro-inflammatory cytokines can augment the expression of the potent procoagulant tissue factor that triggers thrombosis in plaques that undergo rupture of the fibrous cap (Quillard et al., 2017). In this study, after 8 weeks, lipid content, MMPs, inflammatory factors, and systemic inflammation levels were at their highest, which may promote plaque rupture. Thus, we hypothesized that the occurrence of plaque rupture was the result of the atherosclerotic pathophysiological state (such as plaque lipid accumulation, inflammatory reaction, and autophagy levels), vascular remodeling, and systemic inflammation.

Macrophages have traditionally commanded considerable attention due to their fundamental roles in every aspect of plaque formation, development, and rupture (Quillard et al., 2017). Plaque macrophages reflect plaque inflammation and play a role in lipid accumulation as well as in the disruption of the fibrous components of the plaque inducing a more vulnerable plaque phenotype prone to plaque rupture (Libby et al., 2014). Newby (2016) reported that MMPs produced from macrophages can contribute to plaque growth and rupture in atherosclerosis. Thus, the rise of the rate of plaque ruptured may be due to the increased infiltrating macrophages and macrophage autophagy levels.

miRNAs have garnered extensive attention in recent decades. Numerous studies have shown that miRNAs play an important regulatory role in the development and progression of atherosclerosis, by regulating the transcription of atherosclerosis-related genes and by post-transcriptional gene regulation (Feinberg and Moore, 2016; Karunakaran and Rayner, 2016; Gregoli et al., 2017). In order to investigate the underlying mechanism of plaque rupture from miRNA expression levels, we prepared miRNA microarrays after R + C surgery at intervals of 0-, 2-, 4-, 8-, and 12 weeks. We then performed bioinformatics analyses of the microarrays at three levels: atherosclerotic plaque (4- and 12-week) vs. control groups, plaque rupture group (8-weeks) vs. atherosclerotic plaque groups (4- and 12-weeks), and time series level.

In atherosclerotic plaque vs. control groups, 22 union set genes were identified. miR-322-5p (upregulated) and miR-206-3p (downregulated) had the highest degrees of change in expression in the ceRNA regulatory network. miR-322-5p had interactions with Kcnq1ot1 and *Mapk8* to form Kcnq1ot1–miR-322-5p–*Mapk8* ceRNA. A recent study reported that loss of *Mapk8* (also known as *Jnk1*) protects macrophages from apoptosis in low-density lipoprotein receptor null mice, which accelerates early atherosclerosis (Babaev et al., 2016). miR-206-3p can form Kcnq1ot1–miR-206-3p–*Igf1* ceRNA. miR-206 is involved in the PI3K–Akt signaling pathway by regulating *Igf1. Igf1* signaling plays an important role in maintaining atherosclerotic plaque stability due to its effects on vascular smooth muscle cell phenotype (Der Thusen et al., 2011). One study has reported that the activated PI3K–Akt signaling pathway can promote the aggregation of inflammatory cells to accelerate the development of atherosclerosis (Morello et al., 2009). Selective inhibition of the PI3K–Akt signaling pathway can regulate autophagy of macrophage and vulnerability of atherosclerotic plaque (Zhai et al., 2014). Taken together, we speculated that the Kcnq1ot1–miR-322-5p–*Mapk8* and Kcnq1ot1–miR-206-3p–*Igf1* axes may be involved in the formation of atherosclerotic plaque.

Eleven miRNAs were identified in the plaque rupture group vs. atherosclerotic plaque groups. Therein, miR-466h-5p had the highest degree of expression in the ceRNA network, which was enriched in the apoptosis processes of multiple species by regulating *Bcl2*, an important regulator of programmed cell death pathways. Previous studies have reported that there are relative numbers of vascular smooth muscle cells and macrophages in unstable plaques, and apoptosis of vascular smooth muscle cells can lead to plaque rupture (Bennett, 1999). Additionally, macrophage apoptosis is also associated with plaque instability. It has been reported that macrophages are the main cellular components of advanced plaques, the apoptosis of which results in the release of pro-inflammatory factors, further promoting the death of macrophages (Thorp and Tabas, 2009). The phagocytosis and clearance of apoptotic macrophages is insufficient in advanced lesions, and the apoptotic macrophages eventually develop secondary necrosis. Accumulation of necrotic debris leads to plaque instability and thrombosis (Seimon and Tabas, 2009). Thus, we speculated that miR-466h-5p may promote atherosclerotic plaque rupture via apoptosis-related pathways.

For the other 10 miRNAs in plaque rupture group vs. atherosclerotic plaque groups, miR-144-5p has been reported to participate in the occurrence and development of atherosclerosis by regulating the apoptosis of endothelial cells (Fu et al., 2020). miR-450a-5p was found to be dysregulated in atherosclerotic rabbit (Zhang et al., 2018). Sun et al. (2020) suggested that miR-186-5p was involved in the development of atherosclerosis via targeting the PTEN/PI3K/AKT pathway. Additionally, miR-124-5p has been reported to serve as a therapeutic target for proliferative vascular diseases, like atherosclerosis (Lu and Kakkar, 2014). The role of the other miRNAs in atherosclerosis has not been reported to our knowledge. Among all of these miRNA, miR-466h-5p was the most significantly increased miRNA in our study. Thus, we chose it as the critical marker and target in plaque formation and rupture.

In the ceRNAs of the time series module, miR-15a-5p and miR-15b-5p represented the two highest expression levels, both of which regulate *Vegfa* expression and can form ceRNA with Kcnq1ot1. A recent study demonstrated that miR-15a-5p can reduce the inflammation of arterial tissues and vascular endothelial cells, and promote lipid metabolism in diabetic atherosclerotic rats (Liu et al., 2019). miR-15b-5p is reported to be involved in angiogenesis in zebra fish models (Chan et al., 2013). Additionally, miR-15b can decrease plaque stability (Haver et al., 2010). It has been reported that *Vegfa* is a key molecule to enhance angiogenesis and plays a critical role in vascular development (Ylä-Herttuala et al., 2007). Heinonen et al. (2013) have suggested that *Vegfa* can promote the formation of atherosclerotic lesions in ApoE-deficient mice. Taken together, we speculated that miR-15a-5p and miR-15b-5p may be involved in processes from formation to rupture of atherosclerotic plaque by regulating *Vegfa*.

There were several major limitations of the model and ApoE knockout mice. First, detailed local shear stress was not difficult to be analyzed in this model since using micro-ultrasound imaging systems was extremely difficult because of the size of mice carotid arteries and rapid lesion protrusion in the lumen. Second, the existence of plaque neovascularization, which might be important for intraplaque hemorrhage, was not examined in this model. Third, it is also not clear what role the endothelium plays in plaque disruption in this model. Last, for ApoE knockout, although the lesion distribution in ApoE knockout mice was similar to that of humans, the lipoprotein metabolism differed from humans. In mice, the plasma cholesterol is carried by very-low-density lipoprotein and chylomicron particles, whereas in humans, it is mainly transported by low-density lipoprotein (Oppi et al., 2019). In conclusion, the constructed R + C vulnerable plaque model demonstrates almost all pathological manifestations of human atherosclerotic lesions, with a high incidence of plaque rupture with thrombosis. mRNA microarray analysis suggested that miR-322-5p and miR-206-3p may be associated with the formation of atherosclerotic plaque. miR-466h-5p may promote atherosclerotic plaque rupture via apoptosis-related pathways. miR-15a-5p and miR-15b-5p may be involved in processes from formation to rupture of atherosclerotic plaque by regulating *Vegfa*. These DEmis may serve as attractive therapeutic targets for vulnerable plaque rupture. Further experimental studies are needed to validate the role of these miRNAs as therapeutic targets for plaque rupture in animal models.

## Data Availability Statement

The original contributions presented in the study are included in the article/Supplementary Material, further inquiries can be directed to the corresponding author/s.

## Ethics Statement

The animal experiments were approved by the Ethics Committee of Renji Hospital affiliated to Shanghai Jiaotong University School of Medicine.

## Author Contributions

PN and FY designed the research and drafted the manuscript. FW acquired the data. JP analyzed and interpreted the data. SJ revised the manuscript for important intellectual content. All authors read and approved the final manuscript.

## Conflict of Interest

The authors declare that the research was conducted in the absence of any commercial or financial relationships that could be construed as a potential conflict of interest.
